# Generation of transgene-free genome-edited carrot plants using CRISPR/Cas9-RNP complexes

**DOI:** 10.1007/s00299-025-03499-6

**Published:** 2025-04-29

**Authors:** Rajesh Yarra, Patrick J. Krysan

**Affiliations:** Department of Plant and Agroecosystem Sciences, University of Wisconsin-Madison, Madison, Wisconsin 53706 USA

***Key Message*** **We report a process by which transgene-free, gene-edited carrot plants can be efficiently produced by introducing Cas9 ribonucleoprotein complexes into protoplasts and regenerating carrot plants from those protoplasts.**

Carrot (*Daucus carota* L. subsp. *sativus* Hoffm., 2*n* = 2*x* = 18) is an important world vegetable crop and a major source of dietary pro-vitamin A. Recent progress in new plant breeding techniques has facilitated the development of new crop varieties with desired traits in a short period of time (Campa et al. 2024). Among these techniques, the CRISPR/Cas9 (clustered regularly interspaced short palindromic repeats/CRISPR-associated protein) genome editing approach holds significant promise for revolutionizing crop improvement and food production (Wang and Doudna 2023). However, one challenge to use genome editing in crop breeding is the generation of transgene-free gene-edited plants (Kocsisova and Coneva 2023; Gao 2021). Typically, one of the two strategies are employed to achieve transgene-free edited plants. First, a stably integrated CRISPR/Cas9 expression construct used for editing can be removed through genetic segregation. Second, cells can be transiently exposed to editing reagents (Kocsisova and Coneva 2023; Gao 2021). One drawback to use genetic segregation to achieve transgene-free edited plants is that this process is time consuming (Kocsisova and Coneva 2023; Gao 2021). As an alternative to stable integration of Cas9 expression constructs into the genome of the plant, one can use various strategies to transiently expose plant cells to Cas9 and single guide RNAs (sgRNAs). One way to do this is to use polyethylene glycol (PEG)-mediated transfection to deliver Cas9 expression constructs, in vitro-transcribed Cas9-encoding RNA, or preassembled CRISPR/Cas9 ribonucleoproteins (RNPs) to protoplasts (Cardi et al. 2023; Zhang et al. 2021). Tissue culture techniques are then used to regenerate edited plants from the protoplasts. Ribonucleoproteins (RNPs) composed of Cas9 protein and synthetic sgRNAs have been delivered into protoplasts for transgene-free genome editing in various plants (Cardi et al. 2023). While PEG-mediated delivery of Cas9/sgRNA RNPs into carrot protoplasts has been previously reported (Klimek-Chodacka et al. 2021), the regeneration of edited carrot plants using Cas9/sgRNA RNPs has not been reported to date.

Here, we report the efficient production of transgene-free gene-edited carrot plants by transfecting protoplasts with Cas9/sgRNA RNPs. For these experiments, the target gene for editing was the single copy acid soluble invertase isozyme II gene (GenBank accession number Y18706.1). The acid soluble invertase isozyme II is responsible for the breakdown of sucrose into free sugars such as fructose and glucose in the storage roots of carrot. Inactivation of the acid soluble invertase isozyme II in carrot by gene editing would be expected to cause sucrose accumulation in the edible tap root.

To produce null alleles of the carrot acid soluble invertase isozyme II gene, we designed two sgRNAs sgRNA1 (5^/^-TCACTGGTACGATGTTAACG-3^/^) and sgRNA2 (5^/^-GGGTGTTGCACGCGGTCCAT-3^/^) that target exon 3 of this gene (Fig. 1a). After validating the editing efficiency of these two sgRNAs using transient protoplast assays, we conducted two independent editing experiments. In these experiments, we transfected Cas9/sgRNA RNPs, assembled with either sgRNA1 or sgRNA2, into carrot protoplasts and subsequently regenerated the plants. The sgRNA molecules were synthesized by IDT (Coralville, IA, USA) at the 2 nmol synthesis scale. The synthesized sgRNAs were resuspended in nuclease free-IDTE buffer (1X TE buffer, pH 7.5) to a concentration of 100 μM. RNP complexes were preassembled in vitro by gently mixing 200 pmol of sgRNA (2ul of the 100 µM stock), 20 μg (2 μL) of Cas9-GFP protein (10 μg/μl) (IDT, Coralville, IA, USA), and 2 μL of 1X PBS buffer (pH 7.4). This RNP complex (total volume of 6 μl) was incubated at room temperature for 10 min before being used to transfect protoplasts. The assembled RNP complexes for sgRNA1 or sgRNA2 (6μL) were transfected separately into carrot protoplasts resuspended in 200 μL MMG solution (4 mM MES hydrate (pH 5.7), 0.4 M mannitol, 15 mM MgCl_2_) (Meyer et al. 2022) at a concentration of 8.0 × 10^5^ protoplasts per ml. To the protoplast-RNP mixture, 206 μL of freshly prepared 40% PEG solution was slowly added and mixed gently by pipetting. Following 15 min of incubation at room temperature, 4 ml of W5 solution (2 mM MES hydrate (pH 5.7), 154 mM NaCl, 125 mM CaCl_2_, 5 mM KCl) (Meyer et al. 2022) was gently added to the protoplast-RNP-PEG mixture and mixed carefully. The samples were then centrifuged for 4 min at 100 g at room temperature. After centrifugation, the protoplast pellet was resuspended in 10 ml of protoplast culture media (CPP) (Meyer et al. 2022). Subsequent protoplast culture and regeneration of carrot plants was performed according to our previously established protocol (Yarra and Krysan 2024; Meyer et al. 2022).

**Fig. 1:**
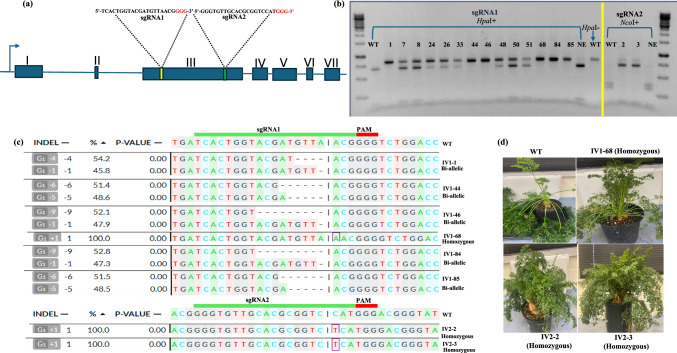
CRISPR/Cas9-RNP-mediated genome editing in carrot plants. **a** Intron–exon structure of the invertase isozyme II gene of carrot and positions of sgRNA1 and sgRNA2. The red letters in the sgRNA sequences indicate the protospacer adjacent motif (PAM) sequences. **b** Restriction digestion pattern of PCR amplicons from wild type, edited and non-edited lines after digestion with *Hpa*I for sgRNA1 (IV1-1,7,8,24,26,33,44,46,48,50,51,68,84, and 85) and with *Nco*I for sgRNA2 (IV2-2 and IV2-3)**.** WT indicates wild-type plant; NE indicates non-edited lines regenerated from protoplasts. “*Hpa*I + ” and “*Nco*I + ” indicate samples treated with those restriction enzymes; “*Hpa*I-” indicates samples were not treated with restriction enzyme. **c** DECODR (Bloh et al. 2021) analysis of Sanger sequencing reads from edited lines homozygous for biallelic for mutations. Sanger sequencing electropherograms for wild type and each edited line are provided in the supplementary information. The sgRNA targeted regions are indicated by a green shaded bar, and the PAM regions are indicated by the red shaded bar. **d** Wild type (WT) and three edited homozygous lines (IV1-68, IV2-2, and IV2-3)

A total of 81 and 31 plants were regenerated from protoplasts transfected with the RNP complexes with sgRNA1 and sgRNA2, respectively. To test for gene editing, genomic DNA was isolated from leaf tissue of regenerated plants with well-established shoots and roots. The targeted region was amplified using the pair of primers (5^/^-ACAAGGGATGGTACCATTTAT-3^/^ and 5^/^-TCTGACATCACTAATTTCGCT-3^/^). Restriction enzyme digestion of the PCR products with *Hpa*I resulted in fragments of 699 bp and 191 bp for wild-type plants (Fig. 1b). Editing at the sgRNA1 target site is expected to disrupt the *Hpa*I recognition site in many cases, and we observed that 14 out of the 81 plants regenerated from the sgRNA1-transfected protoplasts produced *Hpa*I-resistant PCR products. In the case of sgRNA2, editing at this target site is expected to disrupt a *Nco*I recognition site in many cases. Of the 31 plants regenerated from sgRNA2-transfected protoplasts, 2 produced *Nco*I-resistant PCR products, indicative of editing (Fig. 1b).

The editing rate observed in the population of regenerated carrot plants produced from protoplasts transfected with sgRNA1 complexes was 17.28% (14 out of 81 lines), whereas it was 6.45% (2 out of 31 lines) for the sgRNA2 targeted region. Sanger sequencing of the PCR products and analysis of the Sanger traces using DECODR software (Bloh et al. 2021) indicated that three of the edited lines were homozygous for mutations (IV1-68, IV2-2, and IV2-3), five were biallelic (IV1-1, IV1-44, IV1-46, IV1-84, and IV1-85), and eight lines were either heterozygous or chimeric for mutations in the invertase gene (Fig. 1c). The Sanger sequence traces obtained from plants with homozygous edits produce clean sequencing reads that are straightforward to interpret. In cases where biallelic or heterozygous mutations are present, the Sanger sequencing traces appear to be messy at the point where the mutations start due to overlapping reads caused by sequencing two different templates in the same sequencing reaction. To predict the specific mutations present in these biallelic lines, the DECODR web tool (Bloh et al. 2021) was used to analyze of the Sanger DNA sequencing traces. The predicted DNA sequence of each edited allele in the presumed biallelic lines is shown in Fig. 1c. The identified mutations at the targeted regions are single base insertions, or small deletions ranging from 1 to 9 nt (Fig. 1c). The Sanger sequencing traces for wild type and all of the homozygous and biallelic edited lines is provided in supplementary file 1.

Here, we report the production of transgene-free, edited carrot plants from protoplasts by utilizing Cas9/sgRNA RNP transfection. For these experiments protoplasts were transfected with two different sgRNAs targeting the acid soluble invertase isozyme II gene. The edited plants displayed normal growth and development (Fig. 1d). Future work will explore the phenotypic consequence of these mutations on sugar metabolism in the carrot plants. The use of this Cas9-RNP approach to edit agronomically important genes in the carrot genome demonstrates the potential utility of this method for creating transgene-free genome-edited carrot plants.

## Supplementary Information

Below is the link to the electronic supplementary material.

Supplementary file1 (AB1 259 KB)

Supplementary file2 (AB1 255 KB)

Supplementary file3 (AB1 262 KB)

Supplementary file4 (AB1 252 KB)

Supplementary file5 (AB1 260 KB)

Supplementary file6 (AB1 259 KB)

Supplementary file7 (AB1 257 KB)

Supplementary file8 (AB1 255 KB)

Supplementary file9 (AB1 261 KB)

Supplementary file10 (AB1 266 KB)

Supplementary file11 (AB1 255 KB)

Supplementary file12 (AB1 253 KB)

Supplementary file13 (AB1 253 KB)

Supplementary file14 (AB1 254 KB)

Supplementary file15 (AB1 255 KB)

Supplementary file16 (AB1 254 KB)

Supplementary file17 (AB1 254 KB)

Supplementary file18 (AB1 254 KB)
